# Supplementary material for: The Stickland Reaction Precursor trans-4-Hydroxy-l-Proline Differentially Impacts the Metabolism of Clostridioides difficile and Commensal Clostridia
Source: mSphere. 2022 Mar 30;7(2):e00926-21. doi: 10.1128/msphere.00926-21 (PMC9044972; doi:10.1128/msphere.00926-21)
Supplement: TABLE S1 [file msphere.00926-21-st001.docx]

**Primers used to construct vectors for gene deletions and complementation’s in *C. difficile* 630Δerm∆pyrE**

| YH-P253 | atggcgcgccTGGAATGGATATTATTTAGGGGTG | 5' hypD CD630_32820 region - Fwd, AscI for pMTL-YN3 |
| --- | --- | --- |
| YH-P254 | CTATTATCTCATCTTGTAGTGTTCTACTGTGTGGTTCTGCATTAATACTTTC | 5' hypD CD630_32820 region insert - Rev, SOE for pMTL-YN3 |
| YH-P255 | GAAAGTATTAATGCAGAACCACACAGTAGAACACTACAAGATGAGATAATAG | 3' hypD CD630_32820 region insert - Fwd, SOE for pMTL-YN3 |
| YH-P256 | aatcctgcaggACCCCATTACTGCTTGAG | 3' hypD CD630_32820 region insert - Rev, SbfI for pMTL-YN3 |
| YH-P257 | aatggcgcgccCGATATGGATACATTGCTTAAAGC | 5' proC CD630_32810 region insert - Fwd, AscI for pMTL-YN3 |
| YH-P258 | CTCATGTCTTTAGACTTATCTATACAAGCCCCTGAACCAATAAATCCTAAAG | 5' proC CD630_32810 region insert - Rev, SOE for pMTL-YN3 |
| YH-P259 | CTTTAGGATTTATTGGTTCAGGGGCTTGTATAGATAAGTCTAAAGACATGAG | 3' proC CD630_32810 region insert - Fwd, SOE for pMTL-YN3 |
| YH-P260 | aatcctgcaggCCGCCATTTGGATAAAGTAC | 3' proC CD630_32810 region insert - Rev, SbfI for pMTL-YN3 |
| YH-P229 | catggagatctcgaggAGATAGAGACTACATGGTAAAGTGTAG | hypD promoter insert - Fwd, Gibson into pMTL-YN1C |
| YH-P230 | cttgccatGATTTTTCACCCCTAAATAATATC | hypD promoter insert - Rev, Gibson into pMTL-YN1C |
| YH-P231 | gtgaaaaatcATGGCAAGAGGAACTTTTG | hypD CD630_32820 insert - Fwd, Gibson into pMTL-YN1C |
| YH-P232 | cttgcatgtctgcaggCTAGAATGTTTGCTCAGTTCTTC | hypD CD630_32820 insert - Rev, Gibson into pMTL-YN1C |
| YH-P233 | catggagatctcgaggAGATTAATTATGAGCTAACTTTCTAAATAG | proC with promoter CD630_32810 insert - Fwd, Gibson into pMTL-YN1C |
| YH-P234 | cttgcatgtctgcaggTTATTTACTCATGTCTTTAGACTTATC | proC with promoter CD630_32810 insert - Rev, Gibson into pMTL-YN1C |

**Primers used to confirm *C. difficile* 630Δerm mutants using PCR amplification and Sanger sequencing**

| YH-P295 | GACAAAATAATAACAAATATAGTATTATGTAATTG | hypD KO in C.diff 630Δ erm seq primer - Fwd | WT = 4.5kb, KO = 2.2kb, Ta = 60C |
| --- | --- | --- | --- |
| YH-P296 | CAGCTTCTATAGTAGTTCCTGCTG | hypD KO in C.diff 630Δ erm seq primer - Rev | WT = 4.5kb, KO = 2.2kb, Ta = 60C |
| YH-P297 | AACACTAGATATATACAAGGTGTTGG | proC KO in C.diff 630Δ erm seq primer - Fwd | WT = 2.9kb, KO = 2.2kb, Ta = 58C |
| YH-P298 | CTTACACTATATTTGTATATTATCTTGGC | proC KO in C.diff 630Δ erm seq primer - Rev | WT = 2.9kb, KO = 2.2kb, Ta = 58C |
| Cdiff hypD F | ATGGCAAGAGGAACTTTTGAGAGAAC | hypD complementation in C.diff 630Δerm seq primer - Fwd | KO = 0kb, Complemented = 2.4kb, Ta = 62C |
| Cdiff hypD R | CTAGAATGTTTGCTCAGTTCTTCC | hypD complementation in C.diff 630Δerm seq primer - Rev | KO = 0kb, Complemented = 2.4kb, Ta = 59C |
| Cdiff proC F | ATGAAAACTTTAGGATTTATTGGTTCAGG | proC complementation in C.diff 630Δerm seq primer - Fwd | KO = 0kb, Complemented = 0.8kb, Ta = 60C |
| Cdiff proC R | TTATTTACTCATGTCTTTAGACTTATCTATAC | proC complementation in C.diff 630Δerm seq primer - Rev | KO = 0kb, Complemented = 0.8kb, Ta = 59C |

**qRT-PCR primers**

| Gene | Forward | Reverse |
| --- | --- | --- |
| 630-hypD | GCA AGA CAA ATG GCA GAA GAA G | CTG GTT TGT GAG CTG GTA CA |
| 630-proC | GGG AAG GAA TGT CAG CTC TTT | TGA TAC TAT CTC TGC CTC ACC A |
| Chir-hypD | GGT GAA GTT ATG GGT GCT AGT C | GCT GTT GGT CCG TTA GTA TCT |
| Chir-proC | CCA AGA CCT CAG GCT TAC AAA | AGC TGG AGA GCA AAC CAT ATC |
| Chyl-hypD | CTA CAC TCG GTG TGA ACT TCT G | CGG ATA CAA ACG GTC CTA CAT C |
| Chyl-proC | CCG AAC CTT CTG CAG TTC TT | GAA GTA TGG CGC AGG CTA TT |
| Csci-hypD | CTG GAA TGC CTG TGG GTA AA | GGC GAA ATC CGT ATA GGT ACT G |
| Csci-proC | TCA GGT GAC AGA CAG CAA TAA G | GCC TGG AGC GAT CGT AAT AAT |
