# Supplementary figures and images for: The Stickland Reaction Precursor trans-4-Hydroxy-l-Proline Differentially Impacts the Metabolism of Clostridioides difficile and Commensal Clostridia
Source: mSphere. 2022 Mar 30;7(2):e00926-21. doi: 10.1128/msphere.00926-21 (PMC9044972; doi:10.1128/msphere.00926-21)

A

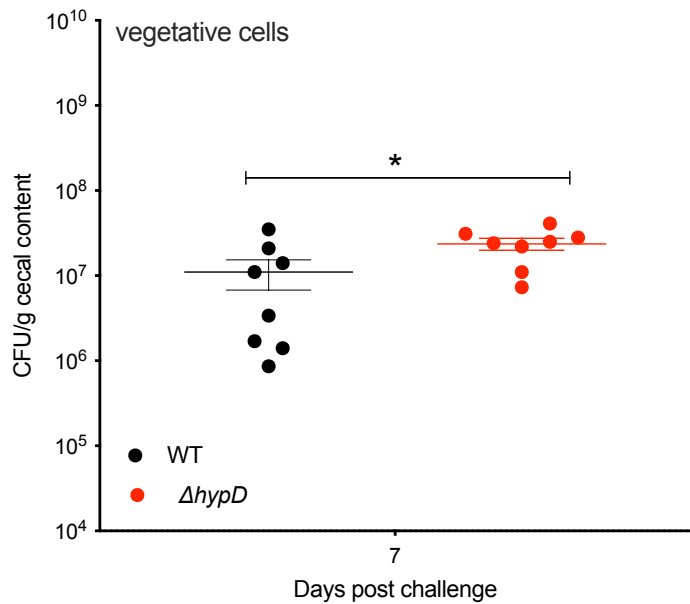

B

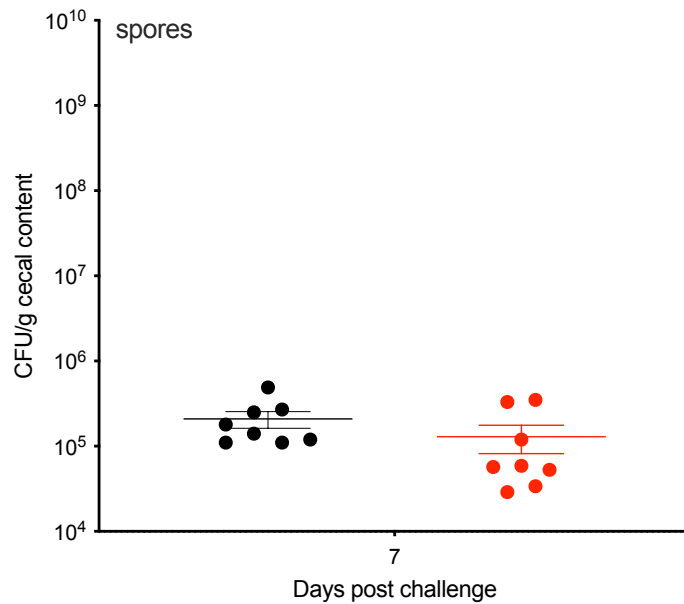

Supplement: FIG S1 [file msphere.00926-21-sf001.pdf]

A

### Alpha Diversity - Family Only

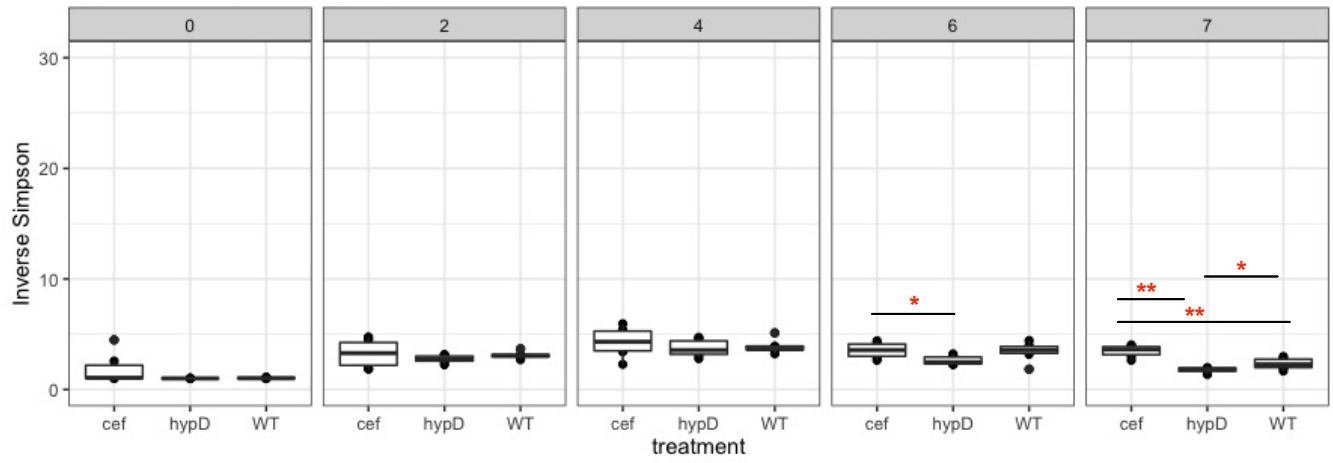

B

### NMDS - All

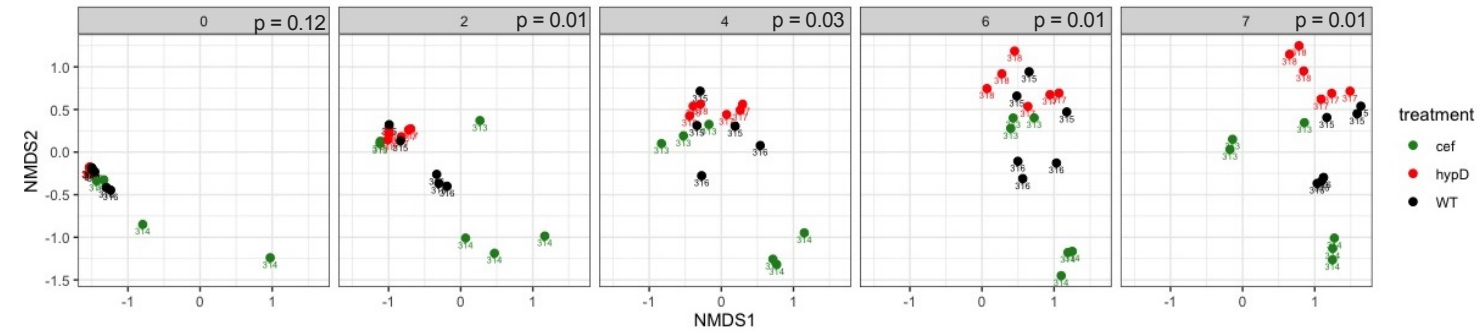

C

### NMDS - Infected

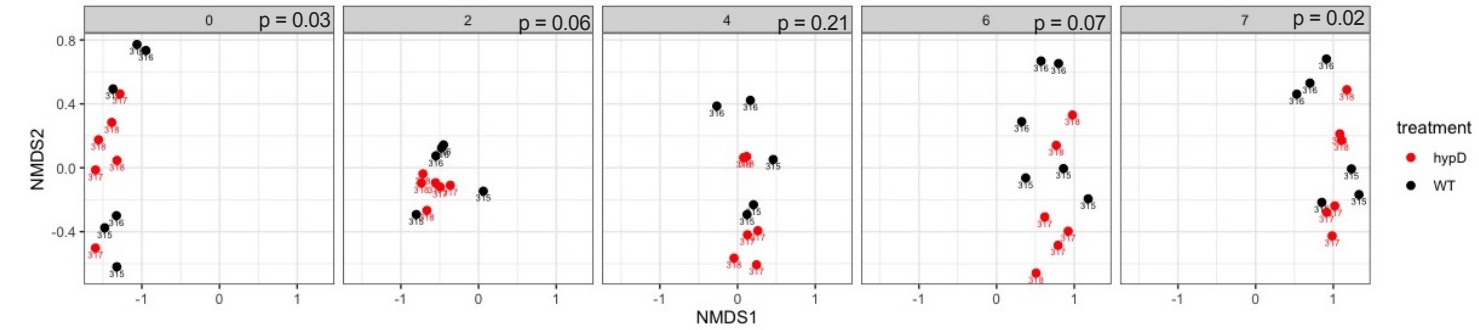

Supplement: FIG S2 [file msphere.00926-21-sf002.pdf]

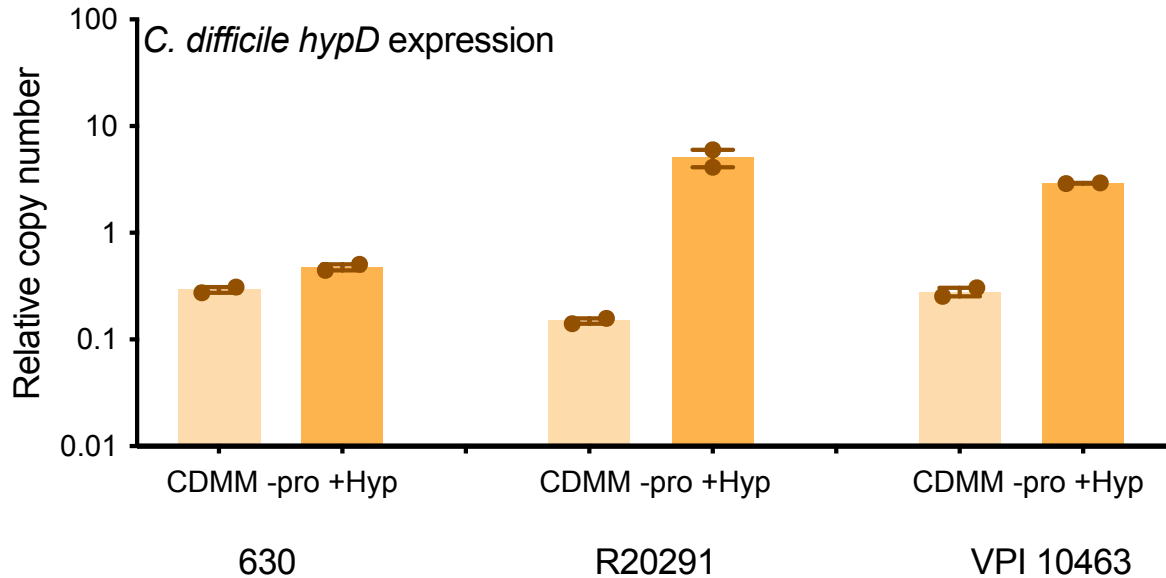

Supplement: FIG S3 [file msphere.00926-21-sf003.pdf]
