# Supplementary material for: The Stickland Reaction Precursor trans-4-Hydroxy-l-Proline Differentially Impacts the Metabolism of Clostridioides difficile and Commensal Clostridia
Source: mSphere. 2022 Mar 30;7(2):e00926-21. doi: 10.1128/msphere.00926-21 (PMC9044972; doi:10.1128/msphere.00926-21)
Supplement: FIG S4 [file msphere.00926-21-sf004.pdf]

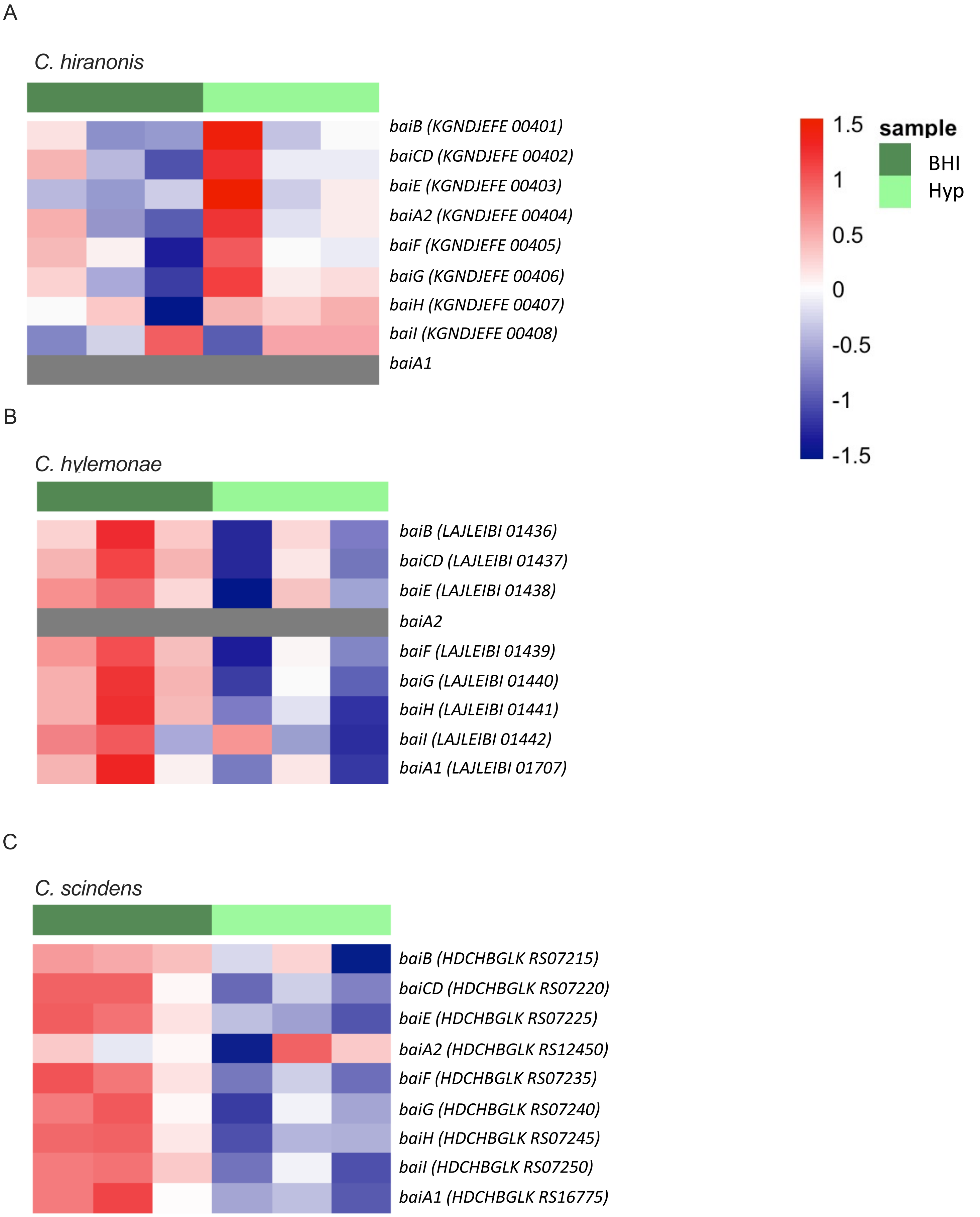
A
C. hiranonis
baiB (KGNDJEFE 00401)
baiCD (KGNDJEFE 00402)
baiE (KGNDJEFE 00403)
baiA2 (KGNDJEFE 00404)
baiF (KGNDJEFE 00405)
baiG (KGNDJEFE 00406)
baiH (KGNDJEFE 00407)
baiI (KGNDJEFE 00408)
baiA1
1.5
1
0.5
0
-0.5
-1
-1.5
sample
BHI
Hyp
B
C. hylemonae
baiB (LAJLEIBI 01436)
baiCD (LAJLEIBI 01437)
baiE (LAJLEIBI 01438)
baiA2
baiF (LAJLEIBI 01439)
baiG (LAJLEIBI 01440)
baiH (LAJLEIBI 01441)
baiI (LAJLEIBI 01442)
baiA1 (LAJLEIBI 01707)
C
C. scindens
baiB (HDCHBGLK RS07215)
baiCD (HDCHBGLK RS07220)
baiE (HDCHBGLK RS07225)
baiA2 (HDCHBGLK RS12450)
baiF (HDCHBGLK RS07235)
baiG (HDCHBGLK RS07240)
baiH (HDCHBGLK RS07245)
baiI (HDCHBGLK RS07250)
baiA1 (HDCHBGLK RS16775)
